# Antiproliferative Effect and the Isolated Compounds of *Pouzolzia indica*

**DOI:** 10.1155/2013/342352

**Published:** 2013-05-09

**Authors:** Chanyapat Sangsuwon, Weena Jiratchariyakul, Yaowalak U-pratya, Tanawan Kummalue

**Affiliations:** ^1^Department of Pharmacognosy, Faculty of Pharmacy, Mahidol University, Bangkok 10400, Thailand; ^2^Department of Medicine, Faculty of Medicine Siriraj Hospital, Mahidol University, Bangkok 10700, Thailand; ^3^Department of Clinical Pathology, Faculty of Medicine Siriraj Hospital, Mahidol University, Bangkok 10700, Thailand

## Abstract

Previous report showed the high potent antiproliferative effect of the methanolic part extracted from the aerial parts of *Pouzolzia indica* on NB4 and HT93A acute leukemic cell lines with the IC_50_ values of 28.5 and 49.8 **μ**g/mL, respectively. The bioassay-guided fractionation of the methanolic part gave 5 fractions, that is, FFI–FFV. FFII, FFIII, and FFIV inhibited the above leukemic cell lines with the IC_50_ values of 15.1 (FFII), 14.4 (FFIII), 32.1 (FFIV), and 31.0 (FFII), 9.7 (FFIII), 10.5 (FFIV) **μ**g/mL, respectively. The compounds in these fractions were isolated using chromatographic technique. FFII contained friedelin **1**, 28-hydroxy-3-friedelanone **2**, and 7-methoxy-coumarin **3**. FFIII contained 6, 7-dimethoxy-coumarin **4**, scopoletin **5**, methyl caffeate **6**. FFIV contained sitosteryl glucoside **7** and a supposed glycosphingolipid **8**. The chemical structures were elucidated by spectroscopic methods.

## 1. Introduction

*Pouzolzia indica* Gaudich.var. *angustifolia* Wedd. (local name “Non tai baihong”) is a Thai medicinal plant in the family Urticaceae [1, 2]. It was used as remedy for the ailments in female infertility, cancer, and inflammation and as emmenagogue and insecticide [3]. The chemical constituents in* P*. *indica* were scarcely reported. Only lanceolone, an isoflavone compounds, was isolated [4]. Previously, the antiproliferative effect of the methanolic part of this plant was reported [5]. It could inhibit the growth of NB4 and HT93A cells with the IC_50_ values of 28.5 ± 0.1 and 49.8 ± 0.7 *μ*g/mL [5], respectively. The apoptosis of NB4 cells treated with 75 *μ*g/mL of this fraction for 24 hours increased from 3.2% to 22.2%, whereas HT93A cells underwent apoptosis from 3.0% to 51.3% when treated with the methanolic part at 150 *μ*g/mL [5]. The previous results, therefore, showed high potent antiproliferative effect of this methanolic part on these acute leukemic cells. From this study, the active extract was further fractionated using column chromatography. The active fractions were determined by bioassay. The compounds in each fraction were isolated and structurally identified.

## 2. Materials and Methods

### 2.1. Cytotoxicity Test

#### 2.1.1. Cell Lines and Culture

NB4 promyelocytic cell line was kindly provided by Ms. Setsuko Miyanishi, Tenri Institute of Medical Research, Japan, and HT93A promyelocytic cell line was kindly provided by Dr. Kenji Kishi, Tokai University, Japan. Long and short types of PML-RAR*α* have been detected in NB4 and HT93A cells, respectively [6–8]. Cells were maintained in RPMI 1640 media supplemented with 10% fetal bovine serum (Stem cell Technology, Vancouver, BC, Canada) with 1% penicillin and streptomycin (Gibco, Life Technologies, Breda, the Netherlands). Cells were incubated at 37°C with 95% humidified atmosphere containing 5% CO_2_ [9].

#### 2.1.2. Cell Viability Assay

Cell viability was assessed using MTT [(3,4,5-dimethylthiazol-2-yl)-2-5-diphenyltetrazolium bromide] assay [10]. In brief, 1 × 10^4^ cells of NB4 and 1 × 10^5^ cells of HT93A were seeded in each well in 96-well plate flat bottom. Cells were treated with each fraction isolated from this medicinal plant for 48 h at the concentrations varying from 0 to 50 *μ*g/mL. After 48 h incubation, 50 *μ*L of 1 mg/mL of MTT in PBS (Sigma, Thailand) was added to each well, and cells were incubated for 4 hr. 100 *μ*L of 10% SDS in 0.01 M HCl was added to stop the reaction and was incubated overnight. The absorbance at 590 nm was measured and read using the ELISA reader (Biorad, USA). Cell viability was calculated using the following formula [11]:

(1) $$\begin{array}{c} \text{Cell}\;\text{viability}\;\left(\%\right)=\left(\frac{\text{sample}\;\text{OD}}{\text{control}\;\text{OD}}\right)\times 100. \end{array}$$

#### 2.1.3. Statistical Analysis

The experiments were performed in triplicate with three independent experiments. Data were expressed as the mean ± standard deviation. The *R*-square equation was used to calculate the IC_50_ value. A *P* value less than 0.05 was considered statistically significance [9].

### 2.2. Phytochemistry

#### 2.2.1. General

^1^H and ^13^C NMR spectra recorded on Bruker DPX-300, Switzerland, with deuterated solvents and TMS as a reference. APCI-MS atmospheric pressure chemical ionization mass spectra were measured on Perkin Elmer mass spectrometer. IR spectra were recorded on FT-IR spectrometer; Perkin-Elmer UV spectra were recorded on Hitachi, U 320 spectrophotometer. Melting points were measured on Digital Electromol 9100. Separation and isolation were performed by column chromatography (CC) using following adsorbents: Diaion HP20, size 250–850 *μ*m, Mitsubishi Chemical Industry, silica gel for CC (63–200 *μ*m, Merck 7734), and low pressure column chromatography (LiChroprep, Merck). TLC: precoated Kieselgel 60 F_254_ (Merck). NP/PEG or NEU spray reagent was used to detect flavonoids and coumarins, 10% H_2_SO_4_ in ethanol was used as universal spray reagent, and 10% FeCl_3_ in ethanol was used to detect phenolic compounds.

#### 2.2.2. Plant Material

The aerial parts of *P. indica* were collected from Ratchaburi Province in the central part of Thailand. The plant was identified by the expert from Forest Herbarium-BKF, Bangkok, Thailand. A plant specimen was deposited with the voucher number of BKF. no. 106441 and SN 096588.

#### 2.2.3. Extraction and Isolation

**Figure 1:**
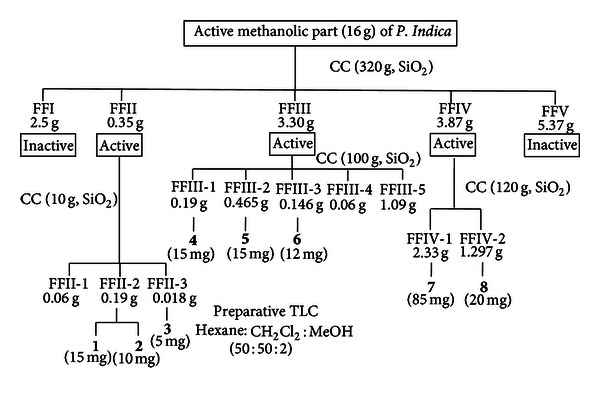
Isolation of compounds **1**–**8** from active methanolic part of *P. indica*.

*P. indica* was extracted with ethanol and fractionated by Diaion HP20 column, eluted with water, water : methanol (1 : 1), methanol, and ethyl acetate. The methanol fraction showed significant antiproliferative effect [5]. This active methanolic part (16 g) was applied on a silica gel column, eluted with gradient solvent systems (ss) of hexane-ethyl acetate and ethyl acetate-methanol, to obtain 5 fractions (FFI–FFV). FFI (2.5 g, ss : hexane-EtOAc, 9 : 1) appeared as oily liquid. FFII (0.35 g, ss : hexane-EtOAc, 1 : 1), FFIII (3.30 g, ss : hexane-EtOAc, 1 : 9), FFIV (3.87 g, ss : EtOAc-MeOH, 8 : 2), and FFV (5.37 g, ss : EtOAc-MeOH, 7 : 3). The bioassay-guided fractionation showed the active fractions, that is, FFII–FFIV. FFII–FFIV were further separated by chromatographic column (Figure 1). FFII was chromatographed on silica gel column, eluted with gradient ss of hexane-acetone and acetone-methanol, to obtain 3 subfractions FFII-1-3. FFII-1 (0.06 g, ss : hexane-Me_2_O, 95 : 5) appeared as oily liquid. FFII-2 (0.19 g, ss : hexane-Me_2_O, 8 : 2) was recrystallized with Et_2_O, and the white needles **1** (15 mg) was obtained. The filtrate was evaporated to dryness and recrystallized with methanol, and **2** (10 mg) was obtained. FFII-3 (0.018 g) was separated on the preparative TLC using solvent system of hexane : CH_2_Cl_2_ : methanol (50 : 50 : 2) giving four separated bands. The band with *R*_*f*_ value of 0.75 was isolated, recrystallized in ethyl acetate resulting **3** (5 mg).

FFIII was chromatographed on silica gel column, eluted with gradient ss of hexane-CH_2_Cl_2_ and CH_2_Cl_2_-methanol, to obtain 5 subfractions FFIII-1–5. FFIII-1 (0.19 g, ss : hexane-CH_2_Cl_2_, 7 : 3) was recrystallized in methanol giving **4** (15 mg). FFIII-2 (0.465 g, ss : hexane-CH_2_Cl_2_, 1 : 1) was recrystallized with methanol giving **5** (15 mg). FFIII-3 (0.146 g, ss : CH_2_Cl_2_-MeOH, 9.5 : 0.5) was recrystallized in methanol giving **6** (12 mg). FFIII-4 (0.06 g, ss : CH_2_Cl_2_-MeOH, 9 : 1) was brown gum. FFIII-5 (1.09 g, ss : CH_2_Cl_2_-MeOH, 8 : 2) appeared as yellowish gum.

**Figure 2:**
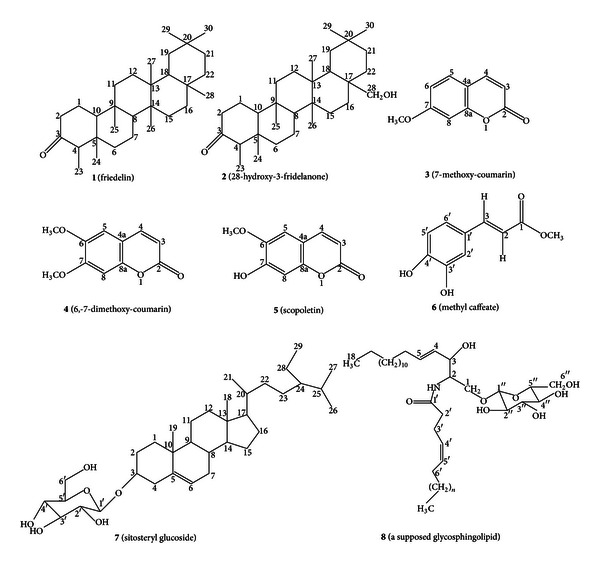
Structures of **1**–**8**.

FFIV was chromatographed on silica gel column, eluted with gradient ss of hexane-acetone and acetone-methanol. It produced 2 subfractions, that is, FFIV-1-2. FFIV-1 (2.33 g, ss : hexane : acetone, 7 : 3) was added with methanol, **7** (85 mg) was precipitated as white powder. FFIV-2 (1.297 g, ss : acetone : methanol, 9 : 1) was added with chloroform : methanol (95 : 5);** 8** (20 mg) was obtained as white powder. Each of the purified compounds **1**–**8** described above possessed the following physicochemical properties and the chemical structures were identified using spectroscopic methods (^1^H NMR, ^13^C NMR, Mass spectra, IR spectra, and UV-spectra). The structure of compounds **1**–**8** (Figure 2) were elucidated as the followings:

(**1**) (friedelin): white needles (Me_2_O). mp 258–260°C. UV (EtOH) *λ*_max⁡_ 220 nm. IR (CHCl_3_) *ν*_max⁡_ 2980, 2927, 2870 (CH), 1706 (C=O), 1462, 1389 (CH) cm^−1^. APCI-MS 427.4156 [M+H]^+^ (calc. C_30_H_50_O 426.7244). ^1^H NMR (CDCl_3_, 300 MHz) *δ* 0.75 (3H, s, H-24), 0.90 (3H, s, H-23), 0.98 (3H, s, H-25), 1.04 (3H, s, H-29) 1.04 (3H, s, H-30), 1.04 (3H, s, H-26), 1.07 (3H, s, H-27), 1.20 (3H, s, H-28), 1.3-1.4 (18H, complex m, H-6, 7, 11, 12, 15, 16, 19, 21, 22), 1.4–1.6 (3H, complex m, H-8, 10, 18), 1.74 (2H, dd, *J *5.5, 3.0 Hz, H-1a, H-1b), 2.2 (1H, m, H-4), 2.34 (1H, m, H-2b), 2.40 (1H, m, H-2a). ^13^C NMR (CDCl_3_, 75 MHz) *δ* 213.1(C3), 59.4 (C10), 58.2 (C4), 53.1 (C8), 42.8 (C18), 42.0 (C5), 41.5 (C6), 41.5 (C2), 39.7 (C13), 39.2 (C22), 38.3 (C14), 37.4 (C9), 36.0 (C16), 35.6 (C11), 35.3 (C19), 35.0 (C29), 32.8 (C21), 32.4 (C15), 32.1 (C28), 31.8 (C30), 30.5 (C12), 30.0 (C17), 28.1 (C20), 22.2 (C1), 20.2 (C26), 18.6 (C27), 18.2 (C7), 17.9 (C25), 14.6 (C24), 6.8 (C23) [12–14].
(**2**) (28-hydroxy-3-friedelanone or canophyllol): white prisms (MeOH). mp 363-364°C. UV (EtOH) *λ*_max⁡_ 220 nm. IR (CHCl_3_) *ν*_max⁡_ 3200–3513 (OH), 1709 (C=O), 2990, 2930, 2855 (C–H), 1466, 1378 (C–H) and 1039, 1116 (C–O). APCI-MS 443.4015 [M+H]^+^ (calc. C_30_H_50_O_2_ 442.5038). ^1^H NMR (CDCl_3_, 300 MHz) *δ* 0.88 (3H, s, H-24), 0.91 (3H, d, *J *7.8 Hz, H-23), 0.99 (3H, s, H-25), 1.02 (3H, s, H-26) 1.01 (3H, s, H-27), 1.19 (3H, s, H-29), 1.23 (3H, s, H-30), 1.3-1.4 (18H, complex m, H-6, 7, 11, 12, 15, 16, 19, 21, 22), 1.4–1.6 (3H, complex m, H-8, 10, 18), 1.96 (2H, dd, *J* 7.6, 3.7 Hz, H-1a, H-1b), 2.32 (1H, m, H-2b), 2.30 (1H, m, H-4), 2.40 (1H, m, H-2a) and 2.5 (2H, m, H-28), 3.71 (1H, 28-OH). ^13^C-NMR (CDCl_3_, 75 MHz) *δ* 213.1 (C3), 68.3 (C28), 59.4 (C10), 58.2 (C4), 53.1 (C8), 42.0 (C5), 41.7 (C22), 41.5 (C2, C6), 39.7 (C13), 39.5 (C18), 38.3 (C14), 37.4 (C9), 35.6 (C11), 35.2 (C17), 34.4 (C29), 33.4 (C19), 32.9 (C30), 31.6 (C21), 31.3 (C16), 30.5 (C12), 30.3 (C20), 29.2 (C15), 23.4 (C1), 19.4 (C25), 19.1 (C26), 18.2 (C7), 18.1 (C27), 14.8 (C24), 6.8 (C23) [13, 14].
(**3**) (7-methoxy-coumarin or herniarin): white needles (MeOH). mp 117-118°C. UV (MeOH) *λ*_max⁡_ 254, 366 nm. IR (CHCl_3_) *ν*_max⁡_ 2927, 2870 (CH), 1682 (C=O), 1533 (C=C) cm^−1^. APCI-MS 176.2015 [M]^+^ (calc C_10_H_8_O_3_ 176.1714). ^1^H NMR (CDCl_3_, 300 MHz) *δ* 7.63 (1H, d, *J* 9.6 Hz, H-4), 6.31 (1H, d, *J* 9.6 Hz, H-3), 7.09 (1H, d, *J* 8 Hz, H-5), 6.95 (1H, s, H-8), 6.90 (1H, d, *J* 8 Hz, H-6) and 3.99 (3H, s, 7-OCH_3_). ^13^C NMR (CDCl_3_ 75 MHz) *δ* 162.02 (C2), 156.3 (C7), 150.0 (C8a), 142.7 (C4), 121.1 (C3), 115.2 (C4a), 113.4 (C6), 111.4 (C8), 109.1 (C5), 56.3 (OCH_3_) [15, 16].
(**4**) (6, 7-dimethoxy-coumarin or scoparone): pale yellow needles (MeOH). mp 146-147°C. UV (MeOH) *λ*_max⁡_ 345 nm. IR (CHCl_3_) *ν*_max⁡_ 2985, 2925, 2855 (CH), 1684 (C=O), 1535 (C=C), 1462, 1389, 1363 and 1311 (CH). APCI-MS 206.1245 [M]^+^ (calc. C_11_H_10_O_4_ 206.1576). ^1^H NMR (CDCl_3_, 300 MHz) *δ*, 7.63 (1H, d,* J* 9.5 Hz, H-4), 6.9 (1H, s, H-5), 6.85 (1H, s, H-8), 6.25 (1H, d,* J* 9.5 Hz, H-3), 3.90 (3H, s, 7-OCH_3_), 3.85 (3H, s, 6-OCH_3_). ^13^C-NMR (CDCl_3_, 75 MHz) *δ* 161.3 (C2), 152.9 (C7), 150.1 (C6), 146.4 (8a), 143.2 (C4), 113.6 (C3), 111.5 (C4a), 108.20 (C5), 100.1(C8), 56.40 (2xOCH_3_) [15, 17].
(**5**) (6-methoxy-7-hydroxy-coumarin or scopoletin): pale yellow needles (CH_2_Cl_2_ : MeOH: 9.5 : 0.5). mp 203-204°C. UV (MeOH) *λ*_max⁡_ 254, 366 nm. IR (CHCl_3_) *ν*_max⁡_ 3400–3550 (OH), 1685 (C=O), 2998, 2938, 2856 (CH), 1589, 1511 (C=C) cm^−1^. APCI-MS 192.2008 [M]^+^ (calc. C_10_H_8_O_4_192.1708). ^1^H NMR (CDCl_3_, 300 MHz) *δ* 7.61 (1H, d,* J* 9.5 Hz, H-4), 6.90 (1H, s, H-5), 6.85 (1H, s, H-8), 6.28 (1H, d,* J *9.5 Hz, H-3) and 3.85 (3H, s, 6-OCH_3_). ^13^C NMR (CDCl_3_, 75 MHz) *δ* 161.5 (C2), 150.2 (C6), 149.7 (C7), 144.0 (C8a), 143.4 (C4), 113.4 (C3), 111.5 (C4a), 107.5 (C5), 103.2 (C8), 56.4 (OCH_3_) [16, 17].
(**6**) ((E)-methyl-3 (3′,4′-dihydroxy-phenyl) acrylate or methyl caffeate): pale yellow crystalline powder (MeOH). mp 157–159°C. UV (MeOH) *λ*_max⁡_ 354 nm. IR (CHCl_3_) *ν*_max⁡_ 3530 (OH), 2995, 2927, 2870 (CH), 1516, 1643 (C=C) and 1690 (C=O). APCI-MS 194.0623 [M]^+^ (calc. C_10_H_10_O_4_ 194.1866). ^1^H NMR (CDCl_3_, 300 MHz) *δ* 7.50 (1H, d, *J* 16.1 Hz, H-3), 7.17 (1H, d, *J* 2 Hz, H-2′), 6.93 (1H, d,* J* 7 Hz, H-5′), 6.88 (1H, dd, *J *7, 2 Hz, H-6′), 6.15 (1H, d,* J* 16.1 Hz, H-2), 3.85 (3H, s, OCH_3_). ^13^C NMR (CDCl_3_-CD_3_OD, 75 MHz) *δ* 168.3 (C1), 147.4 (C4′), 145.5 (C3), 144.8 (C3′), 126.6 (C1′), 121.8 (C2), 115.1 (C2′), 114.1 (C5′), 113.9 (C6′), 51.4 (OCH_3_) [18, 19].
(**7**) (sitosteryl glucoside): white needles (MeOH). mp 258–260°C. UV (EtOH) *λ*_max⁡_ 220 nm. IR (KBr) *ν*_max⁡_ 3200–3450 (OH), 2980, 2927, 2870 (CH), 1560 (C=C). APCI-MS 412.4002 [M − 180]^+^ (calc. C_35_H_60_O_6_ 576.8854. ^1^H NMR (CDCl_3_+CD_3_OD, 300 MHz) *δ* 0.59 (3H, s, H-18), 0.85 (3H, d,* J* 7.3 Hz, H-27), 0.90 (3H, t,* J* 6.5, 6.5 Hz, H-29), 0.91 (3H, s, H-19), 0.92 (3H, d,* J* 7.3 Hz, H-26), 1.12 (3H, d,* J* 7.0 Hz, H-21), 1.30 (2H, m, H-28), 1.74 (1H, m, H-2a), 1.94 (2H, m, H-1), 2.18 (1H, m, H-2b), 2.21 (1H, m, H-4), 3.28–3.5 (5H, m,H-1′, H-2′, H-3′ H-4′, H-5′), 3.50–3.70 (2H, m, H-6′). 3.74 (1H, m, H-3), 5.31 (1H, m, H-6), ^13^C NMR (CDCl_3_+CD_3_OD, 75 MHz) *δ* 140.1 (C5), 121.9 (C6), 100.9 (C1′), 79.0 (C3, C3′), 76.2 (C5′), 75.7 (C2′), 73.4 (C4′), 61.8 (C6′), 56.6 (C20), 55.9 (C17, C24), 50.0 (C9), 45.6 (C8), 42.1 (C13), 40.0 (C12), 39.9 (C4), 37.5 (C1), 36.5 (C10), 33.8 (C7, C22), 31.7 (C14), 29.8 (C2), 29.4 (C16), 29.0 (C25), 28.0 (C23), 24.1 (C15), 23.0 (C28), 22.9 (C11, C21), 20.9 (C26), 19.5 (C27), 19.0 (C19), 11.7 (C29), 11.6 (C18) [16].
(**8**) (a supposed glycosphingolipid): white amorphous powder (CHCl_3_ :  MeOH, 3 : 97). mp 252-254°C (MeOH). UV (EtOH) *λ*_max⁡_ 236 nm, IR (KBr) *ν*_max⁡_ 3230-3450 (OH), 3384 (NH), 2998, 2935, 2851(CH), 1642 (C=O). APCI-MS 751.2596 [M]^+^(calc. C_44_H_81_NO_8_ 751.2539). ^1^H NMR (pyr-d_5_, 300 MHz) *δ* 0.90 (2 × 3H, t, J6, 6 Hz, acyl-CH_3_), 1.20 (2H, m, H-17), 1.30 (complex m, H7-16, (CH_2_)_*n*_), 1.8 (6H, m, H-6, H-3′, H-6′), 2.4 (2H, m, H-2′), 2.6-2.8 (1H, m, OH), 3.90-4.50 (7H, m, H-1′′, H-2′′, H-3′′, H-4′′, H-5′′, H-6′′). 4.24 (1H, m, H-1a), 4.66 (1H,m, H-1b), 4.70 (1H, m, H-5′), 4.75 (2H, m, H-2, H-3), 5.30 (1H, m, H-5, H-4′), 5.50 (1H, dd, J6.6, 1.0 Hz, H-4), 6-8 (4H, br, s, 4xOH), 8.59 (1H, d, J9.2 Hz, NH). ^13^C NMR (pyr-d_5_, 75 MHz) *δ* 175.7 (C1′), 130.9 (C4), 130.7 (C4′), 123.8 (C5), 122.0 (C5′), 105.6 (C1′′), 78.6 (C3′′), 78.4 (C5′′), 75.4 (C2′′), 72.5 (C3), 71.8 (C4′′), 70.5 (C1), 63.0 (C6′′), 57.7 (C2), 34.3 (C2′), 33.3 (C3′), 32.2 (C6, C6′), 29.6 (C7-C16, C7′-(CH_2_)′_*n*_), 22.9 (C17), 14.3 (acyl CH_3_), 14.1 (acyl CH_3_) [ 20-23 ].

## 3. Results

**Figure 3:**
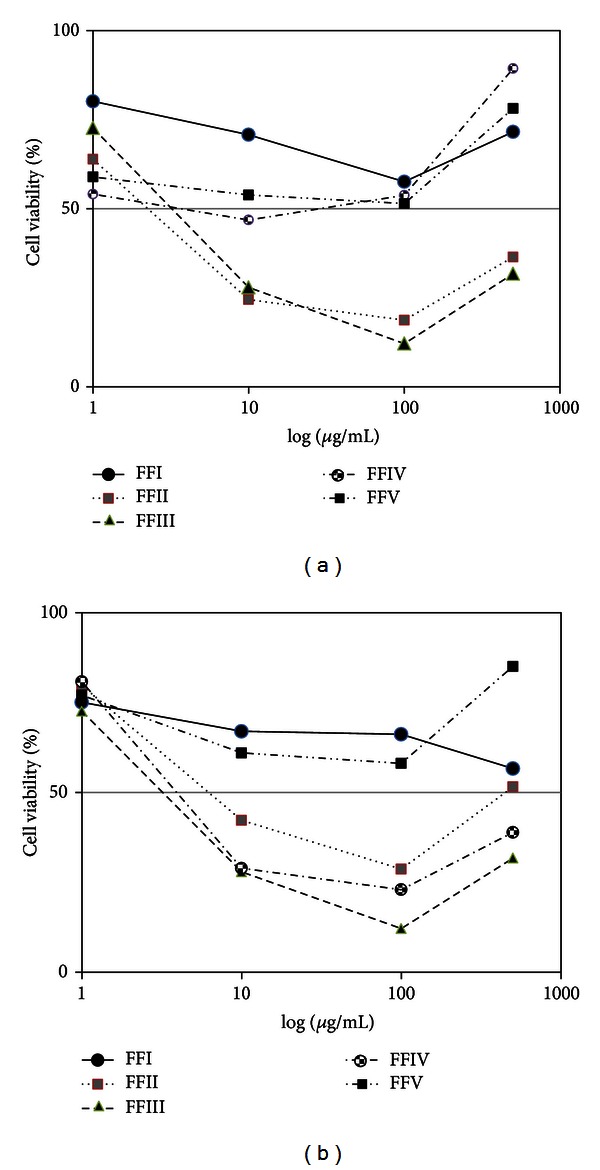
Percentage viable cell of FFI–FFV on leukemic cell lines (a) NB4 and (b) HT93A.

**Figure 4:**
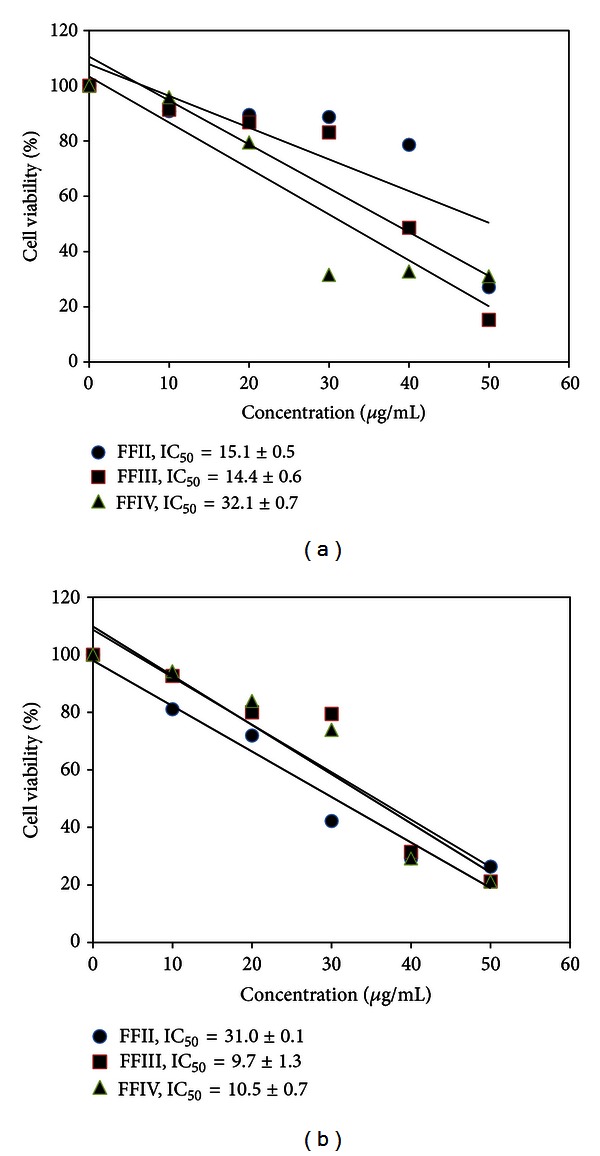
Antiproliferative effect of FFII–FFIV on leukemic cell lines (a) NB4 and (b) HT93A.

The antiproliferative effect of FFI–FFV on human leukemic cell lines was investigated as shown in Figure 3. It was found that FFII, FFIII, and FFIV could inhibit growth of NB4 and HT93A (Figure 4). Therefore, FFII, FFIII, and FFIV were continued to evaluate the IC_50_ values on these cell lines at varying concentrations ranging from 0 to 50 *μ*g/mL. The results showed that FFII, FFIII, and FFIV had the IC_50_ values on NB4 cell line at 15.1 ± 0.5 *μ*g/mL, 14.4 ± 0.6, and 32.1 ± 0.7 *μ*g/mL, respectively, whereas the IC_50_ values of the HT93A cell line were 31.0 ± 0.1, 9.7 ± 1.3, and 10.5 ± 0.7 *μ*g/mL, respectively, as shown in Figure 4. Additionally, FFIII inhibited growth strongly on both NB4 and HT93A cell lines, while FFII inhibits growth strongly on NB4 more than HT93A. FFIV showed strong growth inhibition on HT93A more than NB4.

The active fractions FFII, FFIII, and FFIV were further chromatographed on the silica gel columns repeatedly, and the isolated compounds were identified using spectroscopic methods. FFII was composed of friedelin **1** and 28-hydroxy-3-friedelanone **2** and 7-methoxy-coumarin or herniarin **3**. FFIII was composed of 6,7-dimethoxy-coumarin or scoparone **4**, scopoletin **5**, and methyl caffeate **6**. FFIV was composed of sitosteryl glucoside **7 **and a supposed glycosphingolipid **8**. Sitosteryl glucoside **7** (85 mg) was isolated which was the highest yield as shown in Figure 1.

## 4. Discussion

*P. indica*, which has been long used in Thai traditional medicine for treating various diseases including malignancies, was investigated in this study. Based on our previous report [5], the methanolic part of this plant showed high potent antiproliferative effect on NB4 and HT93A acute promyelocytic cell lines. Here, in this study, we demonstrated that eight compounds were isolated from this active methanolic part. It was chromatographed on CC repeatedly as shown in Figure 1. The bioassay determined the active fractions; they were FFII, FFIII, and FFIV. We found that FFII could inhibit growth on NB4 stronger than HT93A, while FFIII showed growth inhibition on both NB4 and HT93A. Interestingly, FFIV exhibited dominantly growth inhibition on HT93A. The differences in the antiproliferative effects of these fractions might arise from the different active compounds themselves and the interactions with the oncoproteins in these acute promyelocytic cell lines, that is, the long and short types of PML-RAR*α* in NB4 and HT93A, respectively.

The antiproliferative effect of FFII might be caused by the presence of 2 triterpenes, that is, friedelin **1**, and 28-hydroxy-3-friedelanone **2**, and one coumarin **3**, namely, 7-methoxy-coumarin. Previously, the cytotoxicity of 28-hydroxy-3-friedelanone against A549-human lung cancer cell line, LLC-mouse Lewis lung carcinoma, HL60-human promyelocytic cell line, and MCF7-human breast cancer cell line were demonstrated. Hence, some triterpenes could strongly induce apoptosis by attending the mitochondrial membrane potential and regulating the expression of Bcl-2 different compasses [14, 20]. The IC_50_ (*μ*g/mL) of 7-methoxy-coumarin **3** on HL60 and K562 human chronic leukemia cells was also demonstrated with the values of 28.9 and 19.3 *μ*g/mL, respectively [21, 22].

For FFIII, the cytotoxic activity of this fraction might result from coumarins (**4**, and **5**, namely, 6,7-dimethoxy-coumarin, and scopoletin, resp.) including methyl caffeate **6**. The previous reports demonstrated that coumarins could inhibit several human cancer cell lines such as QU-DB large cell lung cancer, and human leukemia HL60 cells [22, 23]. The mechanism of action of coumarins was exerted from the inhibition of tubulin polymerization and the induction of cell cycle arrest at G2/M phase [23]. The involvement of cell cycle inhibition might be due to the inhibition of the release of cyclin D1, an essential enzyme in cell cycle progression [24]. Interestingly, high concentration of scopoletin can have antiproliferative effect on lymphoma cell line by inducing apoptosis [25]. In addition, methyl caffeate can inhibit growth of human cervical adenocarcinoma cell line (HeLa) [26]. Notably, methyl caffeate, which contains 2 hydroxyl groups on aromatic ring, can induce cytotoxic activity via the strong antioxidant activity from these hydroxyl groups [27].

**Figure 5:**
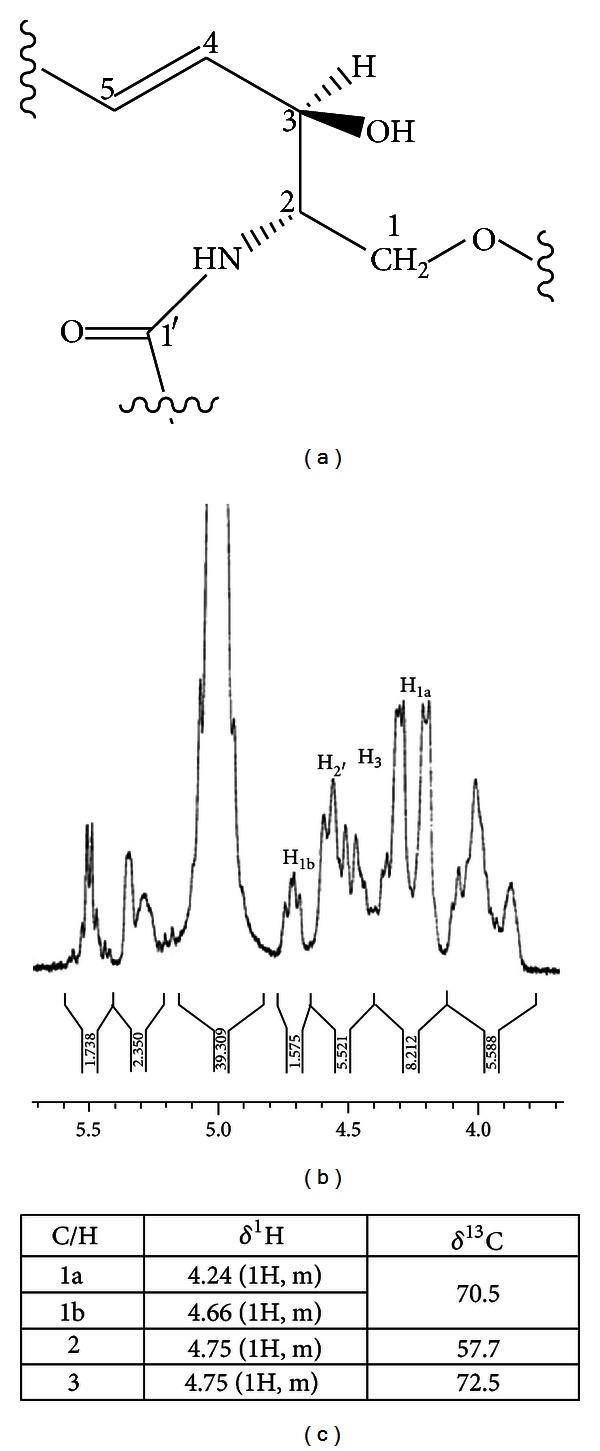
Amino alcohol part of glycosphingolipid (a) chemical structure (b) ^1^H NMR spectrum, and (c) ^1^H and ^13^C assignments.

FFIV inhibited HT93A stronger than NB4 cells. It contained sitosteryl glucoside **7** and a supposed glycosphingolipid **8**. The partial structures of **8** included two acyl chains, one of which was palmitic acid, *β*-*D*-glucose, and an amino alcohol. ^1^H-NMR spectrum of **8** showed that the typical resonances of amino alcohol part of glycosphingolipid were H-1a at *δ* 4.24 (1H, m), H-1b 4.66 (1H, m), and H-2 and H-3 4.75 (2H, m) (Figure 5) [3, 28]. One acyl chain was biosynthetically originated from palmitoyl-CoA which was shown by the long chain methylene protons of **8** appearing as multiplets at *δ* 1.1–1.3 [29]. The presence of sugar protons as complex multiplets at *δ* 3.90–4.50 ppm (7H, m, from H-1′′ to H-6′′) was substantiated by carbon signals at *δ* 105.6 (C1′′), 75.4 (C2′′), 78.6 (C3′′), 71.8 (C4′′), 78.4 (C5′′), and 63.0 (C6′′). The structure of **8** was thus supposed to be a glycosphingolipid. The sitosteryl glucoside **7** was previously reported to have the antiproliferative effect on human colon cancer cell by inducing the apoptotic pathway [27]. The glycosphingolipid **8**, which contains sphingosine, can induce apoptosis involving with the ceramide and sphingosine-1-phosphate-mediated pathway [30, 31]. The result from our study pointed out that coumarins were promising anticancer agent [32]. The extract fraction containing mainly coumarins like FFIII could be developed as a drug material for anticancer phytopharmaceutical.

## 5. Conclusion

The methanolic part of *P. indica* extract inhibited the acute promyelocytic leukemia cell lines, NB4, and HT93A. The bioassay-guided fractionation of the active part got three different active fractions. They were FFII, FFIII, and FFIV. The FFII showed strong growth inhibition on NB4, whereas the FFIII exhibited strong growth inhibition on both NB4 and HT93A. The FFIV demonstrated strong growth inhibition on HT93A. The active compounds isolated from the FFII contained mainly triterpenoids (friedelin **1** and 28-hydroxy-3-friedelanone **2**) and some coumarins (7-methoxy-coumarin **3**). The FFIII contained mainly phenolic compounds (scoparone **4**, scopoletin** 5**, and methyl caffeate** 6**), and the FFIV contained mainly glycosides (sitosteryl glucoside** 7** and glycosphingolipid **8**). *P. indica* was the first report about antiproliferative effect on human leukemic cell lines, and the structures of compounds **1**–**8** were elucidated. The further investigation including drug development will be studied on these fractions especially the FFIII which demonstrated the best antiproliferative effect on both human leukemic cell lines (NB4 and HT93A).

## References

[1] Smitinand T Thai Plant Names (Botanical Names Vernacular Names).

[2] Bennett JJ Plantae Javanicae Rariores.

[3] Srisapoomi T, Jiratchariyakul W, Partkaittikul N, Kummalue T (2008). Effect of two Thai herbal remedies on the sensitivity of chemotherapeutic agents in human cancer cells. *Asian Journal of Traditional Medicines*.

[4] Sayeed A, Sattar MA (2003). A prenylated isoflavone from *Pouzolzia indica*: its in vitro antimicrobial activity and cytotoxicity evaluation. *Oriental Journal of Chemistry*.

[5] U-pratya Y, Jiratchariyakul W, Kummalue T (2008). Anti-proliferative effects of *Pouzolzia indica* on acute promyelocytic cell lines: NB4 and HT93A. *Asian Journal of Traditional Medicines*.

[6] Lanotte M, Martin-Thouvenin V, Najman S, Balerini P, Valensi F, Berger R (1991). NB4, a maturation inducible cell line with t(15;17) marker isolated from a human acute promyelocytic leukemia (M3). *Blood*.

[7] Nishimura N, Furukawa Y, Sutheesophon K (2003). Suppression of ARG kinase activity by STI571 induces cell cycle arrest through up-regulation of CDK inhibitor p18/INK4c. *Oncogene*.

[8] Kishi K, Toba K, Azegami TA (1998). Hematopoietic cytokine-dependent differentiation to eosinophils and neutrophils in a newly established acute promyelocytic leukemia cell line with t(15;17). *Experimental Hematology*.

[9] Skehan P, Storeng R, Scudiero D (1990). New colorimetric cytotoxicity assay for anticancer-drug screening. *Journal of the National Cancer Institute*.

[10] Mosmann T (1993). Colorimetric assay: MTT based for cell proliferation. *Journal of Immunological Methods*.

[11] Kummalue T, O-charoenrat P, Jiratchariyakul W (2007). Antiproliferative effect of *Erycibe elliptilimba* on human breast cancer cell lines. *Journal of Ethnopharmacology*.

[12] Mahato SB, Kundu AP (1994). ^13^C NMR spectra of pentacyclic triterpenoids—a compilation and some salient features. *Phytochemistry*.

[13] Thi Thuy T, Huy Cuong N, Van Sung T (2007). Triterpenes from *Celastrus Hindsii Benth*. *Journal of Organic Chemistry*.

[14] Thao NT, Hung TM, Lee MK, Kim JC, Min BS, Bae K (2010). Triterpenoids from *Camellia japonica* and their cytotoxic activity. *Chemical Pharmaceutical Bulletin*.

[15] Ma CH, Ke W, Sun ZL (2006). Large-scale isolation and purification of scoparone from *Herba artemisiae scopariae* by high-speed counter-current chromatography. *Chromatographia*.

[16] Intekhab J, Aslam M (2009). Constituents from *Feronia limonia*. *Analele Universitii din Bucureti Chimie*.

[17] Kupriyanova GS (1997). NMR studies of the electronic structure of coumarins. *Journal of Structural Chemistry*.

[18] De Carvalho MG, De Carvalho GJA, Braz-Filho R (2000). Chemical constituents from *Ouratea floribunda*: complete ^1^H and ^13^C NMR assignments of atranorin and its new acetyl derivative. *Journal of the Brazilian Chemical Society*.

[19] Lee SP, Jun G, Yoon EJ, Park S, Yang CH (2001). Inhibitory effect of methyl caffeate on Fos-Jun-DNA complex formation and suppression of cancer cell growth. *Bulletin of the Korean Chemical Society*.

[20] Uto T, Sakamoto A, Tung NH (2013). Anti-proliferative activitied activities and apoptosis induction by triterpenes derived from *Eriobotryo japonica* in Human Leucemia cell lines. *Internatioanl Journal of Molecular Science*.

[21] Lacy A (2004). Studies on coumarins and coumarin-related compounds to determine their therapeutic role in the treatment of cancer. *Current Pharmaceutical Design*.

[22] Thanh PN, Jin W, Song G, Bae K, Kang SS (2004). Cytotoxic coumarins from the root of *Angelica dahurica*. *Archives of Pharmacal Research*.

[23] Nardes K, Zahra M, Mohammad R, Nasrollah E, Abbas G (2012). Umbelliprenin is cytotoxic agent QU-DB large cell lung cancer cell line but anti-proliferative against A549 adenocarcinoma cells. *DURA Pharmaceutical Sciences*.

[24] Reyes-Chilpa R, Estrada-Muñiz E, Ramírez Apan T (2004). Cytotoxic effects of mammea type coumarins from *Calophyllum brasiliense*. *Life Sciences*.

[25] Manuele MG, Ferraro G, Barreiro Arcos ML, López P, Cremaschi G, Anesini C (2006). Comparative immunomodulatory effect of scopoletin on tumoral and normal lymphocytes. *Life Sciences*.

[26] Fiuza SM, Gomes C, Teixeira LJ (2004). Phenolic acid derivatives with potential anticancer properties—a structure-activity relationship study—part 1: methyl, propyl and octyl esters of caffeic and gallic acids. *Bioorganic and Medicinal Chemistry*.

[27] Roussi S, Winter A, Gosse F (2005). Different apoptotic mechanisms are involved in the antiproliferative effects of 7*β*-hydroxysitosterol and 7*β*-hydroxycholesterol in human colon cancer cells. *Cell Death and Differentiation*.

[28] Jiratchariyakul W, Moongkarndi P, Okabe H, Frahm AW (1998). Investigation of anticancer components from *Murdannia loriformis* (Hassk.). *Thai Journal of Phytopharm*.

[29] Rimpler H, Arzneistoffe B Georg Thieme.

[30] Ogretmen B, Hannun YA (2004). Biologically active sphingolipids in cancer pathogenesis and treatment. *Nature Reviews Cancer*.

[31] Woodcock J (2006). Sphingosine and ceramide signalling in apoptosis. *IUBMB Life*.

[32] Darla Mark M, Rajesh Kumar K, Bakthavatchala R, Suresh R (2012). Designing, synthesis, and characterization of some novel coumarin derivatives as probable anticancer drugs. *Medicinal Chemistry Research*.

